# Fueling Recovery: The Therapeutic Role of Ketogenic Diet in Neurological Pathologies

**DOI:** 10.7759/cureus.68697

**Published:** 2024-09-05

**Authors:** Allah Yar Yahya Khan, Muhammad Asadullah Khalid Rana, Haseeb Mehmood Qadri, Arham Amir

**Affiliations:** 1 General Surgery, Akhtar Saeed Medical and Dental College, Lahore, PAK; 2 General Surgery, Aziz Fatimah Medical and Dental College, Faisalabad, PAK; 3 Neurosurgery, Punjab Institute of Neurosciences, Lahore, PAK; 4 General Surgery, Shaikh Zayed Medical Complex, Lahore, PAK

**Keywords:** brain, cancer, epilepsy, fat, keto diet, ketogenic, migraine, spine, traumatic brain injury, tumor

## Abstract

The ketogenic diet (KD) has gained a lot of attraction in the management of neurological disorders. KD therapies are high-fat, low-carbohydrate diets intended to shift energy consumption and metabolism from carbohydrates to fat in ketogenesis. The oxidative phosphorylation of ketone bodies generates energy packets for body cells, especially the central nervous system, replacing the role of glucose. KD can benefit multiple neurologic disorders like migraine, motor neuron disease, autism, multiple sclerosis, neuro-oncology, drug-resistant epilepsy, and neurotrauma. KD can provide significant adjuncts to limited conventional therapies, highlighting the feasibility, safety, and potential efficacy in neurology and neurosurgical disease management.

## Editorial

The ketogenic diet (KD), often abbreviated as the “keto diet," is emerging as a promising therapeutic approach for neurologic disorders. KD is characterized by a well-balanced combination of high fat, adequate protein, and low carbohydrate. It creates a metabolic state of ‘nutritional ketoses’ [1]. Various combinations of KD are available and tailored according to the needs of the individual. Proven effects of KD have been demonstrated in the amelioration of weight loss, diabetic state, and hyperglycemia. Amidst the increasing interest in alternative dietary interventions for neurologic recovery, it is important to understand the nuanced role of the keto diet in potentiating clinical practice and guiding future research endeavors.

KD fluctuates excitatory and inhibitory neurotransmitter activities, equilibrates pro-oxidant and antioxidant phenomena, and transitions gut microbiome [1]. KD can play a positive role in neurological recovery by contrasting their metabolism, inflammation, and pathological gene transcription. Positive factors of KD include its limited toxicity, low cost, and easy application. With the increasing prevalence of neurologic disorders and the limitations of conventional neurologic treatments becoming increasingly apparent, there is an escalating need to investigate alternate therapeutic options. The limitations of conventional treatments include availability, compliance, accessibility, affordability, and most importantly, therapeutic efficacy.

A ketogenic diet can benefit neurologic disorders like migraine, motor neuron disease, autism, and multiple sclerosis. Non-neurologic uses include psychiatric and metabolic disorders, including obesity, metabolic syndrome, diabetes mellitus, polycystic ovarian syndrome, and non-alcoholic fatty liver disease that might be comorbid in an individual. However, some healthcare professionals favor the concept that excess saturated fat intake can increase cardiovascular risks [2]. A ketogenic diet helps with improved insulin sensitivity and weight loss, hence decreasing neuro-inflammation. This might help with psychiatric well-being, thus prompting an individual to have better compliance with the advice of the physician, leading to an improved prognosis. A classic ketogenic diet with a low intake of fruits and vegetables might predispose to malignancies and shorten lifespan [3]. This selective dietary intake might not be applicable for patients having significant comorbidities. The adjuvant use of the keto diet for the patients can decrease drug load, especially helping old, dependent, irritable, and financially disadvantaged patients.

The current standard holds that better neurological recovery during the acute period will lead to a longer, more active, and independent life for individuals with spinal cord injury. Changes in diet can be the least invasive approach to recovery from neurotrauma. Although complete results of the Demirel et al. trial are awaited, preliminary results suggest that, compared with a standard diet, five weeks of KD improved upper extremity motor function in patients with acute spinal cord [4]. Patel et al. summarized the findings of five types of diets and dietary patterns in their review to discuss modifiable risk factors affecting the outcomes of traumatic brain injury. Diets rich in fiber and nutrients with curbed amounts of sugars and saturated fats confer the highest form of cardiovascular and CNS protection. However, unlike other diets, KD is not recommended beyond six months for long-term use [5]. Hence, the utility of extended use to assist in the management of traumatic brain injury and acute spinal trauma recovery cannot be determined with limited information.

KD and its modifications may help reduce drug-resistant epilepsy in some patients. The mechanisms mediating the dietary effects remain elusive. A mini-narrative review suggested the impact of KD on host microbiota, leading to a proportion of patients with a greater than 50% reduction in seizures [2]. However, many patients did not respond significantly even after months of KD. In addition, a long-term cohort to evaluate the effects of KD on epilepsy is still pending. The central nervous system (CNS) affects bowel functions via norepinephrine secretion. In turn, the gut microbiota has profound effects on the CNS via the vagus nerve and bioactive substances, such as short-chain fatty acids (SCFAs), tryptophan derivatives, and secondary bile acids [1]. The interaction between the brain, gut, and microbiota is seemingly complex. Yet, the effects of KD on a certain proportion of patients with drug-resistant epilepsy are still promising.

Neuro-oncology can affect all ages and can cause significant morbidity and mortality. Bello et al. compared the usage and effects of KD and standard diet in animals with tumors. KD attenuated the expression of pathways mediated by insulin-like growth factor-1 (IGF-1), platelet-derived growth factor (PDGF), and epidermal growth factor (EGFR) frequently overexpressed in gliomas [2]. In addition, KD has a scavenging effect upon the availability of glucose-6-phosphate (G6P), which hinders the progress of glycolysis as well as the enactment of the pentose phosphate shunt. This biochemical cascade, in turn, allows the control of reactive oxygen species (ROS) levels and cellular oxidative stress. The data on KD and epigenetic modification is still an ambiguous topic. This effect of KD, if proven, can help explain the ability to modify the expression of oncogenes and tumor suppressor genes (TSG) [2]. KD can be a positive complement to conventional therapies in the fight against brain tumors, with glioblastoma being the most hostile form of glioma, thus limiting intervention and helping neurosurgeons and neurologists achieve better recovery for their patients with less effort. This can provide benefits to many patients financially and aid them in avoiding stress, hence having a better quality of life. However, more clinical research is required to characterize the ketogenic diet with standards, make this standardized ketogenic diet easily accessible and adoptable by the person in need regardless of constraints of financial and healthcare facilities, and weigh it against other available and potential treatment options belonging to various fields of healthcare, including surgery, radiology, and medicine.

Nevertheless, KD can provide significant adjuncts to limited conventional therapies, as the complexity of the pathogenesis in neurology and neurosurgery means that effective forms of treatment are still lacking. The role of the keto diet and its combinations should be implemented and tailored according to neuropathology and the nature of the injury of patients. More cohort studies are needed to establish the safety and reliability of KD during the recovery phase from acute neurotrauma. Genetic and epigenetic modifications secondary to KD and their effects on neuro-oncology need further studies at the molecular/biochemical level.
